# Coinfections of Novel Polyomavirus, Anelloviruses and a Recombinant Strain of Myxoma Virus-MYXV-Tol Identified in Iberian Hares

**DOI:** 10.3390/v12030340

**Published:** 2020-03-20

**Authors:** Ana Águeda-Pinto, Simona Kraberger, Michael C. Lund, Christian Gortázar, Grant McFadden, Arvind Varsani, Pedro J. Esteves

**Affiliations:** 1 CIBIO/InBio—Centro de Investigação em Biodiversidade e Recursos Genéticos, Universidade do Porto, Campus Agrário de Vairão, 4485-661 Vairão, Portugal; anaagueda@cibio.up.pt; 2 Departamento de Biologia, Faculdade de Ciências, Universidade do Porto, 4169-007 Porto, Portugal; 3 Center for Immunotherapy, Vaccines, and Virotherapy (CIVV), The Biodesign Institute, Arizona State University, Tempe, AZ 85287, USA; simona.kraberger@asu.edu (S.K.); grantmcf@asu.edu (G.M.); 4 The Biodesign Center for Fundamental and Applied Microbiomics, Center for Evolution and Medicine and School of Life sciences, Arizona State University, Tempe, AZ 85287, USA; mclund2@asu.edu; 5 SaBio Instituto de Investigación en Recursos Cinegéticos IREC-CSIC-UCLM-JCCM, Ronda de Toledo, 28005 Ciudad Real, Spain; Christian.Gortazar@uclm.es; 6 Structural Biology Research Unit, Department of Clinical Laboratory Sciences, University of Cape Town, Cape Town 7701, South Africa; 7 CITS—Centro de Investigação em Tecnologias da Saúde, IPSN, CESPU, 4585-116 Gandra, Portugal

**Keywords:** Leporidae, Iberian hare, *Lepus granatensis*, myxoma virus, *Anelloviridae*, *Polyomaviridae*, coinfection, Spain

## Abstract

Viruses are ubiquitous in nature; however, very few have been identified in the Leporid species. In the fall of 2018, an outbreak of myxomatosis in Iberian hares (*Lepus granatensis*) was reported in Spain and a novel recombinant myxoma virus strain (MYXV-Tol) was identified. To investigate variability within the recombinant region of the MYXV-Tol and identify any potential viral coinfections, samples (ear, eyelid or vaginal) of Iberian hares were collected from Spain and analyzed. The presence of the recombinant region of the MYXV-Tol was confirmed in six out of eleven samples analyzed. Additionally, a polyomavirus (family *Polyomaviridae*), representing a putative new species, and anelloviruses (family *Anelloviridae*) belonging to two putative species were identified, some as coinfection with the recombinant MYXV-Tol. The two polyomavirus genomes were identified in two hares and share >99% genome-wide identity. Based on the analysis of their large T-antigen, the new polyomavirus clusters in a distant clade from other mammals sharing <64% amino acid identity. A total of 14 anelloviruses were identified, which share 63–99% genome-wide identity. Overall, our results show a coinfection of different DNA viruses in the studied samples and raise awareness regarding the extensive unsampled diversity of viruses in hares.

## 1. Introduction

New molecular tools and sequencing technologies have revolutionized viral detection, enabling a better exploration of viral diversity within various organisms. However, most studies have focused on viruses that are strongly associated with disease. The bias toward the study of viruses associated with disease is clear in the Leporidae family (Lagomorpha order). In fact, most of the research undertaken has been directed toward the highly pathogenic lagoviruses (family *Caliciviridae*), such as the rabbit hemorrhagic disease virus [1,2,3], and the leporipoxviruses (family *Poxviridae*) like the myxoma virus (MYXV) [4,5,6,7]. In a recent study of diseased Iberian hares (*Lepus granatensis*) from the Toledo province in Spain, a novel recombinant MYXV strain was identified (MYXV-Tol; GenBank Accession MK836424) showing, for the first time, that a pathogenic myxoma virus could also infect and cause myxomatosis in species of the *Lepus* genus [8]. Historically, MYXV has evolved in natural *Sylvilagus* hosts, like the South American tapeti and the North American brush rabbit. Nonetheless, when MYXV encounters naïve European *Oryctolagus* rabbits, the virus causes the lethal myxomatosis disease. The genome of the recombinant MYXV-Tol identified in Iberian hares is ~99% similar to MYXV variants/strains previously reported circulating in European rabbits, with the exception of a recombinant region ~2800 bp in length and three disrupted genes (*M009L*, *M036L* and *M152R*). The recombinant region encodes four additional open reading frames (ORFs), which were more closely related, but not identical, to the MYXV proteins encoded by *M060R*, *M061R*, *M064R* and *M065R* genes [8,9,10].

Besides the rabbit hemorrhagic disease virus and MYXV, there is limited information on viruses associated with Leporids. A recent study has reported the presence of a novel herpesvirus (*Herpesviridae* family) in Iberian hare samples collected during the myxomatosis outbreak in Spain [11]. Metagenomics studies targeting hare fecal samples found circular replication-associated proteins encoding single-stranded (CRESS) DNA viruses in the families *Genomoviridae* and *Smacoviridae* as well as unclassified groups associated with the European hare (*Lepus europaeus*) and Snowshoe hare (*Lepus americanus*) [12,13].

To determine whether the Iberian hares found dead in Spain harbor viruses in addition to the MYXV-Tol strain, a metagenomic approach was used to identify circular DNA viruses. Using this approach, we identified circular DNA viruses for which we designed specific abutting primers to screen and recover full genomes from various Iberian hare samples collected in Spain following a myxomatosis outbreak. In these samples, virus genomes belonging to the *Anelloviridae* and *Polyomaviridae* families were identified and recovered. Neither of these have been previously reported in Leporids. The *Anelloviridae* family is a group of highly diverse circular single-stranded DNA viruses [14,15,16,17,18] with genome sizes ranging from ~2–4 kb. First identified in humans in 1997 [15], anelloviruses have been found in a wide range of animals, including pigs, non-human primates, seals, bats and horses [16,18,19,20,21], and are largely host species-specific. Polyomaviruses on the other hand are circular double-stranded DNA viruses of ~4–7 kb that, like anelloviruses, are host specific and have been identified in various animals [22,23,24]. In our analysis of various sample types from 11 hares, we identified 14 viruses belonging to two new species of anelloviruses and two viruses belonging to a new species of polyomavirus. Furthermore, we highlighted that three of the hares were coinfected with the MYXV-Tol variant, and either both or one of the novel anelloviruses and/or polyomaviruses.

## 2. Materials and Methods

### 2.1. Sample Collection and Viral Nucleic Acid Extraction

Samples from eleven symptomatic Iberian hares (19 samples in total from either ear, eyelid or vaginal sample) found dead in the fall of 2018 were collected and stored in RNAlater^®^ (Sigma, St. Louis, MO, USA) at −80 °C, as shown in Table 1. These samples were collected from different regions of Spain: Toledo, Cuenca, Ciudad Real and Madrid. Viral nucleic acid was extracted using 5 g of each sample according to a modified phenol–chloroform extraction protocol [25] described in Águeda-Pinto et al. [8].

### 2.2. Detection of the MYXV-Tol

To detect the presence of the new MYXV-Tol, primers flanking the unique recombinant region (~2800 bp) were designed based on the previously described MYXV-Tol sequence (GenBank Accession MK836424), shown in Table S1, that yielded a ~4.8 kb amplicon. Viral nucleic acid from each individual sample was used as a template for PCR amplification using Kapa HiFi HotStart DNA polymerase (Kapa Biosystems, Wilmington, MA, USA) following manufactures’ recommendations at an annealing temperature of 60 °C. The amplicons were resolved on a 0.7% agarose gel stained with SYBR Safe (ThermoFisher Scientific, Waltham, MA, USA) and ~4.8 kb size fragments were excised, gel purified and cloned into pJET1.2 plasmid vector (ThermoFisher Scientific, Waltham, MA, USA). The resulting recombinant plasmids were Sanger sequenced by primer walking at Macrogen Inc. (Seoul, Korea). The sequence contigs were assembled using Geneious 11.1 [26].

### 2.3. Detection and Recovery of Anelloviruses and Polyomavirus Genomes

Previously, the MYXV-Tol variant from sample Lag01_EL, shown in Table 1, was cultured in permissive rabbit cells (RK13, ATCC # CCL-37), purified and sequenced using high-throughput sequencing, which led to the characterization of the full genome of a new myxoma strain, MYXV-Tol, GenBank Accession MK836424 [8].

In this study, viral DNA from the sample Lag01_EL was extracted using the Roche High Pure Viral nucleic acid kit (Roche Diagnostics, Indianapolis, IN, USA). Circular molecules were amplified using rolling circle amplification (RCA) with the Illustra TempliPhi 100 Amplification Kit (GE Healthcare, Chicago, IL, USA). The RCA amplicons were used to generate a 2 × 100 bp Illumina sequencing library and sequenced on an Illumina HiSeq4000 (Illumina, San Diego, CA, USA) at Macrogen Inc. (Seoul, Korea). The paired-end reads were de novo assembled using metaSPAdes 3.12.0 [27]. Contigs with terminal sequence redundancy were assumed to represent circular molecules and all contigs >750 nucleotides (nt) were analyzed using BLASTx [28] against a viral GenBank RefSeq protein database.

Based on these contigs, abutting primers were designed, shown in Table S1, and these primers were used to screen and recover full genomes from the 19 individual samples of 11 animals by PCR. The total DNA extracted from each of the 19 tissue samples was subjected to RCA to amplify circular molecules and 0.5 µL of this was used as a template with Kapa HiFi HotStart DNA polymerase (Kapa Biosystems, Wilmington, MA, USA) following manufactures’ recommendations at an annealing temperature of 60 °C with specific primers for the amplification of the anellovirus and polyomavirus genomes. The amplicons were resolved on a 0.7% agarose gel stained with SYBR Safe (ThermoFisher Scientific, Waltham, MA, USA). Amplicons (~2.5 kb for anelloviruses and ~5.4 kb for polyomaviruses) were excised, gel purified and cloned into pJET1.2 plasmid vector (ThermoFisher Scientific, Waltham, MA, USA). The resulting recombinant plasmids were Sanger sequenced by primer walking at Macrogen Inc. (Seoul, Korea). The sequence contigs were assembled using Geneious 11.1 [26].

### 2.4. Sequence Analysis of MYXV-Tol Regions, Anelloviruses and Polyomaviruses

#### 2.4.1. MYXV-Tol Recombinant Region Sequences

A dataset of MYXV-Tol recombinant region sequences was assembled with sequences from this study (*n* = 7) and the two available in GenBank (GenBank accession # MK836424 and MK340973). These were aligned using MUSCLE [29] and any polymorphism, insertions or deletions were identified manually. The nucleotide sequence pairwise identities were determined using SDT [30].

#### 2.4.2. Anellovirus Sequences Analyses

A dataset of the most closely related anelloviruses (*n* = 4) was created. The ORF1 sequences were extracted and an alignment of the ORF1 amino acid sequences was used to infer a maximum likelihood phylogenetic tree using PHYML [31] with the WAG+G substitution model, determined as the best fit model using ProtTest [32]. Branches with <0.8 aLRT support were collapsed using TreeGraph2 [33] and the resulting phylogenetic tree was midpoint rooted.

#### 2.4.3. Polyomavirus Sequence Analyses

A dataset of representative polyomaviruses (*n* = 125) was downloaded from GenBank. From these, the large T-antigen and VP1 sequences were extracted. The aligned amino acid sequences of the large T-antigen was used to infer a maximum likelihood phylogenetic tree using PHYML [31] with the rtREV + G + I substitution model, determined as the best fit model using ProtTest [32]. Branches with <0.8 aLRT support were collapsed using TreeGraph2 [33] and the resulting phylogenetic tree was rooted with large T-antigen sequences of fish polyomaviruses [22]. The genome-wide nucleotide, the large T-antigen and the VP1 amino acid sequence pairwise identities were determined using SDT v1.2 [30].

## 3. Results and Discussion

After the outbreak of myxomatosis in Iberian hares, samples from Spain were collected to evaluate the presence of novel DNA viruses. Previously, the MYXV from one of the Iberian hare samples (Lag01; eyelid) was cultured in permissive rabbit cells (RK13, ATCC # CCL-37). Following purification, the genome of the MYXV was sequenced using high-throughput sequencing, which led to the identification of the recombinant region (~2.8 kb) derived from an unknown poxvirus in the MYXV genome and thus the recombinant strain, MYXV-Tol [8]. Following this report, other studies [9,10] also identified the MYXV containing the same novel ~2.8 kb recombinant region.

**Figure 1:**
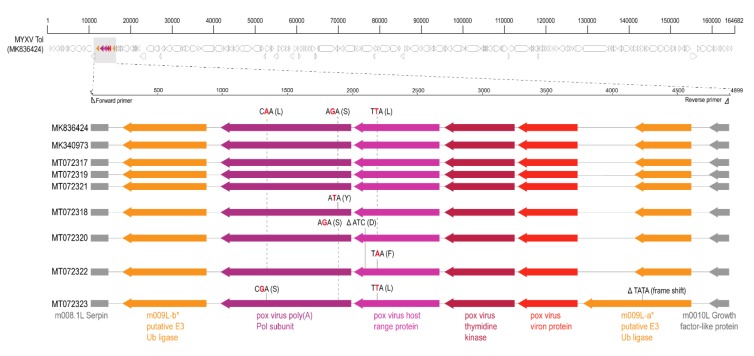
Myxoma virus genome schematic with recombinant region highlighted. The variations identified in the recombinant gene cassette of the sequenced MYXV-Tol variants from various samples are summarized in reference to the MYXV-Tol GenBank accession. SNPs are shown with red highlighted nucleotide and the encoded amino acid in brackets. Deletions are shown with Δ symbol and the deleted amino acid in brackets.

In this study, we identify the recombinant region of MYXV-Tol in seven of the 18 Iberian hare tissue samples from four hares (not including sample Lag01 eyelid from which the full MYXV was isolated). Sequence analyses of the recombinant region show that the obtained amplicons are >99% similar to the recombinant region in the MYXV-Tol (GenBank accession # MK836424) strain previously reported [8]. Among these sequences, three single nucleotide polymorphisms (SNPs) and two deletions were identified, shown in Figure 1. All the SNPs resulted in non-synonymous changes. One deletion of three nucleotides (ATC) located in the pox virus host range gene (GenBank accession # MT072320) resulted in the deletion of an amino acid (D) near the C terminus. In the sequence of the recombinant region of the MYXV-Tol from Lag04 V sample (GenBank accession # MT072323) there was a four nucleotide (TATA) deletion in the m009L gene resulting in a frame shift and extension of the open reading frame.

**Figure 2:**
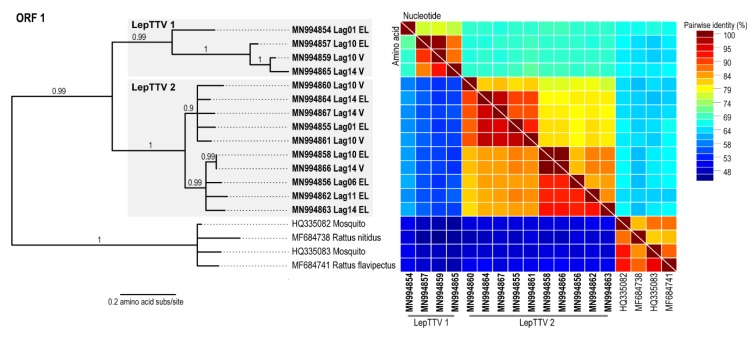
Maximum likelihood phylogenetic tree of the anelloviruses ORF1 protein sequences of those recovered from hares together with those most closely related, available in GenBank. A pairwise comparison color matrix of the ORF1 amino acid and nucleotide sequence is shown to the right of the phylogenetic tree.

In the 19 Iberian hare tissue samples, a diverse range of unique anelloviruses (*n* = 14) were recovered, as seen in Table 1, suggesting that these viruses are common in Iberian hares. The hare- derived anelloviruses have a genome organization that has at least three ORFs and a conserved untranslated region (UTR). The hare anelloviruses range in size from 2490 to 2529 nt. Based on the last report of the International Committee on Taxonomy of Viruses (ICTV) [34], the classification of viruses in the family *Anelloviridae* is based on a global alignment pairwise identity of the ORF1 gene nucleotide sequence, with the cut-off values of >35% divergence for species and >56% divergence for genera. To facilitate the assignment of the anelloviruses at species and genera levels, a nucleotide pairwise comparison was generated, shown in Supplementary Data S1, and based on this, the anelloviruses genomes could be tentatively classified into two species which we refer to as Lepus torque teno virus (LepTTV) 1 and 2 from here on. Of the 14 genomes identified, four form a single LepTTV 1 supported clade, and 10 sequences form a second supported clade comprising of LepTTV 2s, shown in Figure 2. The closest relatives of the anelloviruses identified from hares are those identified in rodents (GenBank accession # MF684738 and MF684741) [35] and mosquitoes (GenBank accession # HQ335083 and HQ335082) [36]. The analysis of the pairwise identity for the ORF1 nucleotide, amino acid and full genome revealed that the LepTTVs share >63%, >56% and >70% pairwise identity with each other, respectively, shown in Figure 2 and Supplementary Data S1. When compared to anelloviruses previously identified in other organisms, LepTTVs share 61–67% (nt), 48–53% (aa), and 61–64% (full genome) pairwise identity. LepTTV 1 was identified in the eyelid (*n* = 2) and vaginal (*n* = 2) samples from three individual hares, shown in Table 1. LepTTV 2 was identified in the ear, eyelid (*n* = 5) and vaginal (*n* = 2) samples from three individual hares, shown in Table 1.

**Figure 3:**
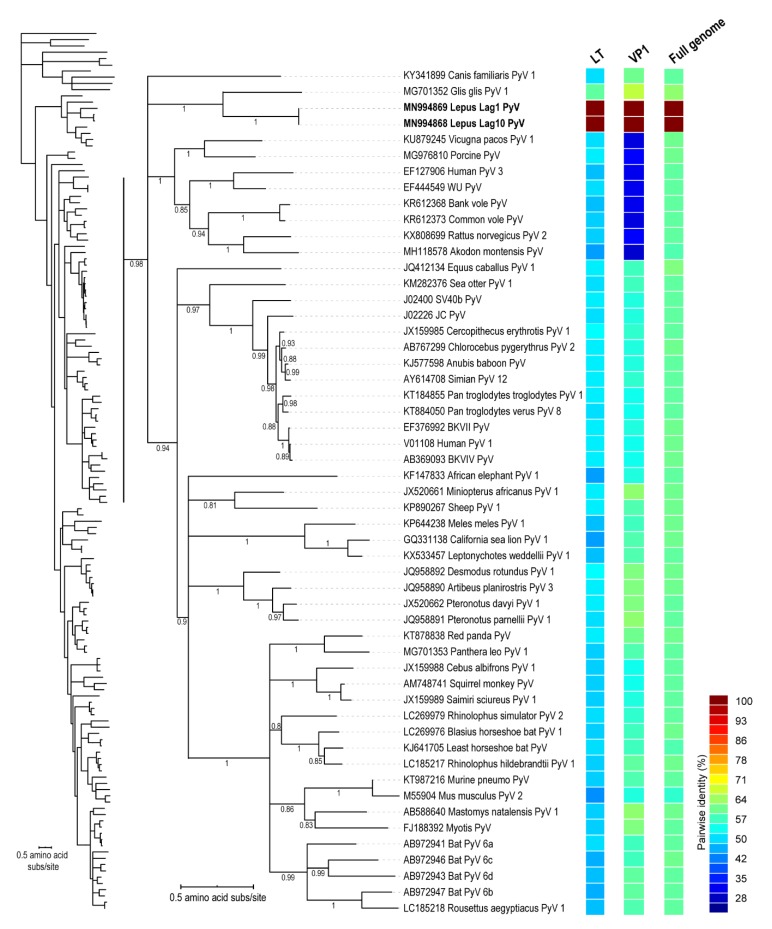
Maximum likelihood phylogenetic tree of the polyomavirus large T-antigen amino acid sequences of polyomaviruses identified in hares and representative sequences of polyomavirus species available in GenBank. The enlarged clade shows those most closely related to the hare polyomavirus. A pairwise comparison color matrix of the large-T protein, VP1 protein and the full genome is provided to the right of the phylogenetic tree.

The polyomaviruses (5386 bp) were detected in two samples, the eyelid of Lag01 and Lag10, shown in Table 1. Typically, polyomavirus genomes are divided into three different regions: a non-coding region that regulates the expression of early and late genes; an early region that encodes the large T-antigen and the small T-antigen; and a late gene region that encodes the capsid proteins VP1, VP2, and VP3 [37,38]. In the genome of the polyomavirus recovered from the Iberian hare samples, we identified all the polyomavirus ORFs. The large T-antigen, VP1 and genome-wide pairwise analysis of the *Lepus* polyomavirus (LepPyV) and those most closely related revealed that these two LepPyV isolates share >99% identity and are most closely related to the *Glis glis* polyomavirus (GenBank accession # MG701352) [39] sharing 60% (large T-antigen), 67% (VP1) and 64% (full genome) pairwise identity, shown in Figure 3. The large T-antigen of the hare-derived polyomaviruses recovered in this study is nested within a larger mammal clade but forms a distinct sub-clade. The species demarcation for polyomaviruses is based on >15% divergence of the large T-antigen, thus the hare-derived polyomavirus represents a new species and we tentatively refer to this polyomavirus as Lepus polyomavirus 1 (LepPyV 1) and this would be part of the *Betapolyomavirus* genus [24,40].

Anelloviruses have a high degree of genetic variability between genotypes and strains and can establish persistent infections in their hosts, with no clear associated pathology [41]. However, it is still poorly understood how the host’s immune system is affected by the continuous exposure to anelloviruses [15,42]. Reports suggest that anelloviruses have a negative impact in several clinically relevant infections by modulating the baseline level of inflammation and interference with molecules of different signal pathways (reviewed in Maggi and Bendinelli [43]). For example, anellovirus ORF2 protein suppresses the NF-κB pathway, a key regulatory element that participates in the synthesis of proinflammatory cytokines like IL-6, IL-8, and cyclo-oxygenase-2 [44]. It was also found that anelloviruses encode miRNAs that could be involved in viral immune evasion and regulation of IFN signaling [45,46]. Studies have shown that the polyomavirus large T-antigen is capable of inactivating some host proteins such as p53, which is responsible for the control of cell-cycle inducing apoptosis [47,48]. Polyomaviruses are also able to prevent lysis of infected cells and initiate oncogenic transformation [38,49,50]. However, without further studies it is not possible to determine whether the coinfections of MYXV-Tol with LepTTV or LepPyV, present in Iberian hares, have any effect on the myxomatosis disease manifestation.

## 4. Concluding Remarks

In this study, we provide the first report of anelloviruses and polyomaviruses associated with hares. In addition, we identify the presence of the MYXV-Tol variant in four hares, and reveal coinfections in three hares, of either both or one of the novel anelloviruses and/or polyomaviruses. Hare populations have declined since the late 1960s which is thought to be in part due to disease [7,51,52]. The recent documentation of an outbreak of myxoma virus in hares caused by a prevalent recombinant MYXV-Tol [8,9,10] highlights another threat to the species. As an important food source for many predator species, such as the endangered Iberian lynx (*Lynx pardinus*), evaluating circulating pathogen populations and monitoring disease spread as well as spillover of pathogens is crucial for the survival of the hare and other species higher on the trophic cascade. Overall, this study expands our knowledge of DNA viruses associated with hares, highlighting the unsampled diversity of viruses, and the importance of investigating pathogen complexes within a host to further understand disease dynamics.

## Figures and Tables

**Table 1:** Sample information of the hare samples analyzed in this study including geographic origin, tissue type and GenBank accession numbers of the viral sequences.

			Poxvirus	Anellovirus		Polyomavirus
Animal ID	Location	Tissue Type	MYXV-Tol	LepTTV1	LepTTV2	LepPyV1
Lag01	Spain: Toledo	Eyelid	MK836424	MN994854	MN994855	MN994868
Lag02	Spain: Cuenca	Ear	-	-	-	-
Lag03	Spain: Ciudad Real	Ear	-	-	-	-
Lag04	Spain: Ciudad Real	Vaginal	MT072323	-	-	-
Lag05	Spain: Ciudad Real	Ear	-	-	-	-
		Eyelid		-	-	-
Lag06	Spain: Ciudad Real	Ear	MT072322	-	-	-
		Eyelid	MT072321	-	MN994856	-
		Vaginal	MT072320	-	-	-
Lag07	Spain: Ciudad Real	Ear	MT072319	-	-	-
		Eyelid	MT072318	-	-	-
Lag10	Spain	Eyelid	-	MN994857	MN994858	MN994869
		Vaginal	-	MN994859	MN994860 MN994861	-
Lag11	Spain: Toledo	Eyelid	-	-	MN994862	-
		Vaginal	-	-	-	-
Lag12	Spain: Ciudad Real	Ear	-	-	-	-
		Eyelid	-	-	-	-
Lag14	Spain: Madrid	Eyelid	-	-	MN994863 MN994864	-
		Vaginal	MT072317	MN994865	MN994866 MN994867	-

## Supplementary Materials

The following are available online at https://www.mdpi.com/1999-4915/12/3/340/s1. Table S1: Summary of primers used to amplify the MYXV-Tol recombinant cassette sequences, and the anellovirus and polyomavirus genomes. Supplementary Data S1: Pairwise comparison of the full genomes of anelloviruses e and the global alignment pairwise identity matrix of the ORF1 nucleotide used for species demarcation.
