# Supplementary material for: Coinfections of Novel Polyomavirus, Anelloviruses and a Recombinant Strain of Myxoma Virus-MYXV-Tol Identified in Iberian Hares
Source: Viruses. 2020 Mar 20;12(3):340. doi: 10.3390/v12030340 (PMC7150814; doi:10.3390/v12030340)
Supplement: Supplementary file 1 [file viruses-12-00340-s001.zip › viruses-741911/Table S1.pdf]

Virus group	Accession #	Fwd 5'-3'	Rev 5'-3'
**MYXV-Tol**	MT072317<br>MT072318<br>MT072319<br>MT072320<br>MT072321<br>MT072322<br>MT072323	TTAAACATAAGACGAGGACCAGATACTTCA	GTAGCATTAAACAATGTTTCACTTAACCC
**LepPyV1**	MN994868<br>MN994869	AGACCAAGGGGAAAAGGAAAGTTTCTCTTG	TGTTATTGGTCCAGCCTCAAGAGATCTAGT
**LepTTV1**	MN994854	GTACTCTGCATCTACATATACCAGGTAGTC	AGAGACTTTAATCTGTGGATGAAACAGTG
	MN994857<br>MN994859<br>MN994865	ATACTGCAAACTGGCCTATGGACCTTTTAT	CTGTTATTGTTTTGAAAGTCACCTGGTGGC
**LepTTV2**	MN994855	CTATATAGTCTAACTGTGGATGTGGCCA	TATACTTCGATCCAGAATACAGGGATTTCG
	MN994856<br>MN994860<br>MN994864<br>MN994867	GTGCTTGGAGGTGGTATAAAGATTTTATGT	AATGAATAGTGGCTGGAATTGGATGAAAGA
	MN994858<br>MN994862<br>MN994863<br>MN994866	TATGAACCTGTTGTTTTTGTACCAGTCTG	CACCTGGGATATGGACCATTCATTTATAAA
	MN994861	GTTATGGGCCGTTTGTCTATAAAAACAAG	CCAGTTTGATGAATCTGTTGTTCTTATACC
